# Tea Polyphenols Regulate Key Mediators on Inflammatory Cardiovascular Diseases

**DOI:** 10.1155/2009/494928

**Published:** 2009-07-19

**Authors:** Jun-ichi Suzuki, Mitsuaki Isobe, Ryuichi Morishita, Ryozo Nagai

**Affiliations:** ^1^Department of Advanced Clinical Science and Therapeutics, Graduate School of Medicine, University of Tokyo, 7-3-1 Hongo, Bunkyo, Tokyo 113-8655, Japan; ^2^Department of Cardiovascular Medicine, Tokyo Medical and Dental University, 1-5-45 Yushima, Bunkyo-ku, Tokyo 113-8519, Japan; ^3^Department of Clinical Gene Therapy, Osaka University, 2-2 Yamada-oka, Suita 565-0871, Japan; ^4^Department of Cardiovascular Medicine, University of Tokyo, 7-3-1 Hongo, Bunkyo, Tokyo 113-8655, Japan

## Abstract

Tea polyphenols known as catechins are key components with many biological functions, including anti-inflammatory, antioxidative, and anticarcinogenic effects. These effects are induced by the suppression of several inflammatory factors including nuclear factor-kappa B (NF-*κ*B). While these characteristics of catechins have been well documented, actions of catechins as mediators on inflammation-related cardiovascular diseases have not yet been well investigated. In this article, we reviewed recent papers to reveal the anti-inflammatory effects of catechins in cardiovascular diseases. In our laboratory, we performed oral administration of catechins into murine and rat models of cardiac transplantation, myocarditis, myocardial ischemia, and atherosclerosis to reveal the effects of catechins on the inflammation-induced ventricular and arterial remodeling. From our results, catechins are potent agents for the treatment and prevention of inflammation-related cardiovascular diseases because they are critically involved in the suppression of proinflammatory signaling pathways.

## 1. Introduction

Green tea is known to prevent cardiovascular diseases; tea consumption is known to be associated with lower mortality of clinical myocardial infarction [1, 2]. It has been reported that green tea consumption reduced cardiovascular disease mortality in the Japanese population [3–5]. Catechins are important components of green tea with many biological functions, including anti-inflammatory, antioxidative, and anticarcinogenic effects [6–9]. These effects are induced by the suppression of several inflammatory factors including nuclear factor-kappa B (NF-*κ*B), a multipotential promoter of matrix metalloproteinases (MMPs), cytokines, and adhesion molecules [10]. The major tea catechins are epigallocathechin-3 gallate (EGCG), epigallocathechin (EGC), epicathechin-3 gallate (ECG). While these characteristics of tea catechins have been well documented [11], their effects on inflammatory cardiovascular diseases have not been well investigated. Recently, we have reported the effects of tea catechins on myocardial ischemia [12], acute myocarditis [13], heart transplantation [14], and atherosclerosis [15]. In this article, we reviewed these reports and other investigations.

## 2. Catechins Suppressed NF-*κ*B in Transplantation

Cardiac transplantation has been established to treat end-stage heart failure, however acute rejection and graft arterial diseases (GADs) are still problems [16–19]. We have revealed that NF-kB plays a key role in the progression of cardiac allograft rejection. To confirm the effects of catechins on rejection, we have used a class II allomismatch combination of the mice to perform cardiac transplantation [20]. The transplanted mice were assigned randomly to two groups. The recipient mice were orally supplemented with tea catechins (20 mg/kg/day, THEA-FLAN 90S (Ito-en Co. Shizuoka, Japan) which includes EGCG: 45.2% ECG: 13.7% EGC: 0.23%) daily. This ratio is the natural dose ratio in the extracts from green tea. For control, transplanted mice were supplemented with normal water without the catechins.

**Figure 1:**
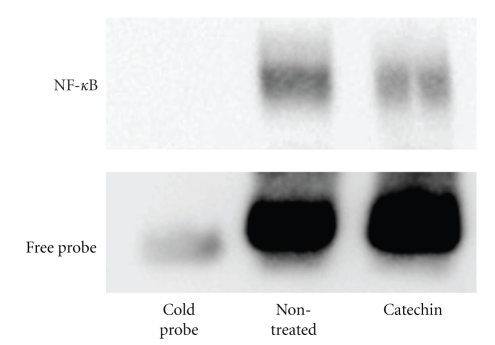
*Gel mobility shift assay of cardiac transplantation. *Gel mobility shift assay documented that increased NF-*κκ*B binding activity was observed in nontreated allografts. This enhanced NF-*κ*B binding was reduced by catechin administration (20 mg/kg/day, THEA-FLAN 90S).

Although severe myocardial cell infiltration and fibrosis was observed in nontreated allografts at day 60, tea catechins markedly attenuated myocardial cell infiltration and fibrosis. Immunohistochemically, enhancement of CD4, CD8, CD11b, intercellular adhesion molecule (ICAM)-1, and vascular cell adhesion molecule (VCAM)-1 expression was observed in nontreated allograft myocardium, infiltrating cells and coronary arteries. However, catechin markedly attenuated expression of all these factors. RNase protection assay was employed to examine expression of cytokine mRNA in hearts. Levels of Th2 cytokine IL-10 was significantly elevated in the catechin-treated group compared with that of nontreated group. To prove the effect of catechins on NF-*κ*B, we performed a gel mobility shift assay using the EMSA kit. The analysis documented that catechin administration reduced NF-*κ*B binding activity (Figure 1) [14]. In the study, we have demonstrated that tea catechins reduced both myocardial remodeling and GAD formation with altered several inflammatory factors via suppression of NF-*κ*B binding activity. Because blockade of NF-*κ*B binding activity results in suppression of cell adhesion molecules, the factors are critical in transplantation because they can induce immunological tolerance [21–24]. Therefore, regulation of NF-*κ*B binding activity by catechins is clinically effective for suppression of transplant rejection.

Recently, Tripathi et al. demonstrated that green tea extract (GTE) in combination with low dose CyA significantly prolongs graft survival as well as increase the production of immunosuppressive cytokines in the murine model of nonvascularized cardiac allografts. They concluded that the potential of GTE as an adjunctive therapy in combination with CyA to prolong allograft survival and to reduce CyA induced nephrotoxicity [25].

## 3. Catechins Regulate MMPs in Myocardial Ischemia

**Figure 2:**
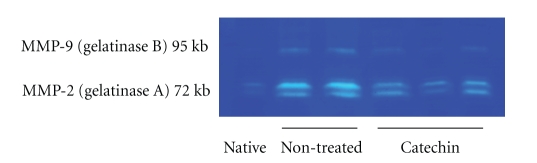
*Zymography of myocardial ischemia.* Gelatin zymography showed that increased gelatinase (MMP-2 and MMP-9) activity was observed in hearts in the non-treated ischemia group. However, this enhanced gelatinase activity was decreased by catechin administration (20 mg/kg/day, THEA-FLAN 90S).

Myocardial ischemia and ventricular remodeling causes significant damage leading to severe heart failure. It has been known that NF-*κ*B related inflammation is enhanced by ischemia and reperfusion of myocardium; the activated NF-*κ*B induces MMPs. Thus, decoy against NF-*κ*B reduces myocardial inflammation induced by ischemia/reperfusion injury [26]. Since MMPs are key components in the positive feedback loop of heart remodeling, the inhibition is an effective therapy for myocardial reperfusion injury [27–30]. It is also well known that catechins suppress several inflammatory factors including MMPs induced by NF-*κ*B [10]. To clarify the role of catechins on the ischemic hearts, we made a rat myocardial ischemia model by ligation of the left anterior descending coronary artery and this was continued for 28 days. After ischemic injury, the nontreated ischemia group showed significant decline of blood pressure compared to non-treated sham-operated group. However, the catechin (20 mg/kg/day, THEA-FLAN 90S) administration suppressed the decline of the blood pressure compared to that of non-treated ischemia group on days 14 and 21. Pathologically, the anterior wall of the hearts was completely fibrotic and remaining area showed interstitial fibrosis and cell infiltration. However, catechin-treated hearts showed significantly less infarct size, infarct length, left ventricular circumference, and left ventricular inner diameter than those in the non-treated ischemia group**.** Immunohistochemically, increased numbers of CD4, CD8, CD11b ICAM-1, and ED-1 positive infiltrating cells were observed in the non-treated ischemia group, while the catechin administration suppressed the numbers significantly. Finally, to reveal the role of MMPs, the infarct region and myocardium were separated under a dissecting microscope and they were used zymography as previously reported. This showed that increased gelatinase (MMP-2 and MMP-9) activity was observed in hearts in the non-treated ischemia group. However, this enhanced gelatinase activity was decreased by catechin administration (Figure 2). In summary of myocardial ischemia, we revealed that catechins prevented ventricular remodeling after ischemic injury due to the suppression of proinflammatory factors including MMPs.

## 4. Catechins Suppress Cytokine Expression in Myocarditis

**Figure 3:**
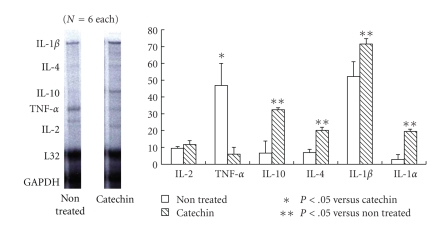
*RNase protection assay of myocarditis.* TNF-alpha mRNA level was markedly decreased in the catechin treated group compared with that of control group. On the other hand, mRNA levels of Th2 cytokines such as IL-4 and IL-10 in the catechin treated group (20 mg/kg/day, THEA-FLAN 90S) were markedly enhanced compared with that of control group.

Myocarditis is a serious disease in clinical settings, patients with myocarditis may present with rapidly progressive heart failure, shock, or arrhythmia. Although acute myocardial inflammation is an essential etiology for the progression, any established treatment has not yet been elucidated [31–35]. Experimental autoimmune myocarditis (EAMs) is a rat model that is characterized by myocardial damages and multinucleated giant cell infiltration. This has been used as a disease model of human acute myocarditis [36–40]. To clarify the effects of catechins on myocarditis, we administered the catechins (20 mg/kg/day, THEA-FLAN 90S) to rats after the induction of EAM. We found that the catechins significantly reduced the heart weight/body weight ratio compared to that of non-treated EAM controls. Echocardiogram revealed the catechins improved the cardiac function compared to the controls. Pathologically, non-treated control EAM animals showed severe myocardial cell infiltration and fibrotic lesions. However, the catechin treatment showed significantly less myocardial cell infiltration and fibrosis areas compared to those in controls. Immunohistochemistry revealed that enhanced expression of CD4, CD8, CD11b, ICAM-1, and NF-*κ*B on infiltrating and arterial endothelial cells was observed in non-treated EAM hearts, while the catechins suppressed the expression. To examine expression of cytokine mRNA in EAM hearts, RNase protection assay was used. TNF-alpha mRNA level was markedly decreased in the catechin-treated group compared with that of control group. On the other hand, mRNA levels of Th2 cytokines such as IL-4 and IL-10 in the catechin-treated group were markedly enhanced compared with that of control group (Figure 3). We revealed that the myocardial cell infiltration, fibrosis, proinflammatory cytokines were enhanced in the EAM progression and the catechins suppressed the development of these changes with altered cytokine expression [13].

## 5. Catechins Altered Adhesion Molecules and Nitric Oxide

**Figure 4:**
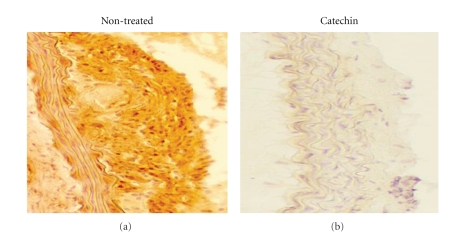
*Immunohistochemistry of atherosclerosis.* Panels show representative immunohistochemical findings. VCAM-1 expression was enhanced in the aortic walls of non-treated LDLRKO mice. However, catechin administration (2% THEA-FLAN 90S contained high fat chaw) suppressed the expression in the organs of LDLRKO mice.

To evaluate the effects of tea catechins for the development of atherosclerosis induced by hyperlipidemia, we administered catechins (2 or 4% THEA-FLAN 90S contained high fat chaw) to LDL receptor knockout (LDLRKO) mice. Immunohistocemically, VCAM-1, a critical adhesion molecule for vascular diseases, expression was enhanced in the endothelial cells, smooth muscle cells, and infiltrating cells in the aortic walls of LDLRKO mice. However, catechin administration significantly suppressed VCAM-1 expression in the atherosclerotic lesions in LDLRKO mice, although LDLRKO mice with the 2% catechins showed comparable cholesterol levels (Figure 4) [15]. In the study, catechins prevent the development with or without changing the plasma lipid levels in the animals through the suppression of adhesion molecules. Babu and Lie reviewed that catechins have further effects on cell adhesion molecules. They showed that catechins prevent vascular inflammation via suppression of leukocyte adhesion to endothelium and subsequent transmigration through inhibition of transcriptional factor NF-*κ*B-mediated production of adhesion molecules both in endothelial cells and inflammatory cells [41].

Nitric oxide (NO) is an important molecule that plays a pivotal role in inflammatory conditions of hearts, and many papers showed interesting data. Babu and Liu demonstrated that catechins regulate vascular tone by activating endothelial NO [41]. Paquay et al. revealed that the catechins are potent peroxynitrite scavengers and are effective inhibitors of inducible NO synthase (iNOS) [42]. Agnetti et al. also evaluated that GTE supplementation counteracted on iNOS induction and activity in cardiomyocytes [43]. It is also noteworthy that EGCG inhibits endothelial exocytosis, the initial step in leukocyte trafficking, and vascular inflammation by increasing Akt phosphorylation, eNOS phosphorylation, and NO production [44].

## 6. Summary and Future Direction

We have demonstrated that the catechin intake significantly suppresses the expression of inflammatory factors including adhesion molecules, cytokines, and MMPs. These key factors are known to be regulated by NF-*κ*B, which is a central mediator for the development of inflammatory diseases. We have reported specific inhibition of NF-*κ*B using a decoy in the myocardial ischemia [26], myocarditis [36], and heart transplant rejection [45]. In these studies, the NF-*κ*B decoy suppresses many inflammatory factors including adhesion molecules, cytokines, and MMPs. Although catechins are not specific inhibitors of NF-*κ*B, they have similar effects to the inhibitors such as suppression of adhesion molecules and other inflammatory factors. Therefore, catechins have the potential to suppress clinical inflammatory diseases.
